# Use of CO_2_-Derived Variables in Cardiac Intensive Care Unit: Pathophysiology and Clinical Implications

**DOI:** 10.3390/jcdd10050208

**Published:** 2023-05-10

**Authors:** Vladimir L. Cousin, Raphael Joye, Julie Wacker, Maurice Beghetti, Angelo Polito

**Affiliations:** 1 Réanimation Pédiatrique, Women, Child and Adolescent Department, Geneva University Hospital, 1205 Geneva, Switzerland; 2 Pediatric Cardiology Unit, Women, Child and Adolescent Department, Geneva University Hospital, 1205 Geneva, Switzerland

**Keywords:** CICU, congenital cardiac abnormalities, veno-arterial CO_2_ difference, VCO_2_/VO_2_ ratio, pediatric

## Abstract

Shock is a life-threatening condition, and its timely recognition is essential for adequate management. Pediatric patients with congenital heart disease admitted to a cardiac intensive care unit (CICU) after surgical corrections are particularly at risk of low cardiac output syndrome (LCOS) and shock. Blood lactate levels and venous oxygen saturation (ScVO_2_) are usually used as shock biomarkers to monitor the efficacy of resuscitation efforts, but they are plagued by some limitations. Carbon dioxide (CO_2_)-derived parameters, namely veno-arterial CO_2_ difference (ΔCCO_2_) and the VCO_2_/VO_2_ ratio, may represent a potentially valuable addition as sensitive biomarkers to assess tissue perfusion and cellular oxygenation and may represent a valuable addition in shock monitoring. These variables have been mostly studied in the adult population, with a strong association between ΔCCO_2_ or VCO_2_/VO_2_ ratio and mortality. In children, particularly in CICU, few studies looked at these parameters, while they reported promising results on the use of CO_2_-derived indices for patients’ management after cardiac surgeries. This review focuses on the physiological and pathophysiological determinants of ΔCCO_2_ and VCO_2_/VO_2_ ratio while summarizing the actual state of knowledge on the use of CO_2_-derived indices as hemodynamical markers in CICU.

## 1. Introduction

Shock and low cardiac output syndrome (LCOS) are life-threatening conditions characterized by an inadequacy between oxygen delivery (DO_2_) and oxygen consumption (VO_2_), leading to cellular dysfunction and end-organ lesions [1]. Timely recognition is crucial for early and aggressive management of such dreadful conditions. Therefore, sensitive biomarkers to assess tissue perfusion and cellular oxygenation are required to guide clinical management and prognosis.

Serum lactate level and central venous oxygen saturation (ScVO_2_) have been used traditionally as markers of tissue perfusion and adequacy of DO_2_ in case of shock. Nonetheless, ScVO_2_ and lactate have drawbacks that need to be known in order to correctly interpret their values in the intensive care setting. Moreover, if their role as predictors of unfavorable outcomes is undeniable, resuscitation algorithms based either on lactate or ScVO_2_ failed to demonstrate clear clinical benefits [2,3,4,5,6,7,8].

The measurement of SvO_2_ (venous oxygen saturation measured in the pulmonary artery) has become a rarity, especially in pediatric patients, as a pulmonary catheter must be placed, which is a complicated and risky procedure. SvO_2_ and ScVO_2_ are closely correlated, provided that the central venous catheter tip is correctly positioned at the junction between the superior venous cava and the right atrium.

ScVO_2_ use is based on the comparison between the VO_2_ and the extraction of oxygen by the tissue, using the Fick formula:

VO_2_ = Cardiac Output × Oxygen Extraction

That is

VO_2_ = Cardiac Output × (CaO_2_ − CvO_2_)

Knowing that CaO_2_ = Hb × 1.34 × SpaO_2_ + 0.003 × PaO_2_ and CvO_2_ = Hb × 1.34 × ScVO_2_ + 0.003 × PaO_2_

And after rearrangement of the equation, the final formula:

ScVO_2_ = SpaO_2_ − VO_2_/[CO × Hb × 1.34]

A reduction of oxygen delivery (the denominator of the equation) will first lead to an increase in oxygen extraction to maintain the VO_2_. As a result, ScVO_2_ will decrease proportionally to the reduction of DO_2_, regardless of the cause that generated the DO_2_ reduction, thus providing a global picture of the patient’s hemodynamic status. The role of ScVO_2_ as a marker of both hemodynamic deterioration and clinical response to treatments has been extensively studied in both adult and pediatric ICU patients [9]. Specifically, after pediatric cardiac surgeries, ScVO_2_ levels have been associated with progressive hemodynamic deterioration and unfavorable clinical outcomes [10,11].

However, several limitations of ScVO_2_ should be mentioned, and its interpretation comes with some caveats. In fact, low ScVO_2_ values indicate a global imbalance between DO_2_ and VO_2_ without giving any further information regarding the individual components of these two elements, such as arterial oxygen saturation, hemoglobin, and CO. Moreover, during the early phase of resuscitation, the increase in DO_2_ may lead to an increase in VO_2_, which could result in a transitory decrease in ScvO_2_ levels. In a situation of shock, a mismatch between DO_2_ and capillary perfusion may lead to a misleadingly normal ScVO_2_ resulting from oxygen extraction impairment [9,12,13].

Serum lactate is a key metabolic parameter that could be related to both hypoperfusion and hypoxia [14]. It is produced in all human cells as part of intracellular glucose metabolism. In patients with shock, DO_2_ could be insufficient to meet cellular metabolism demands. As a consequence, the absence of aerobic mitochondrial metabolism leads to an over-production of lactate from the intracellular excess pyruvate.

As lactate level is a marker of hypoperfusion, serum lactate’s peak level, as well as lactate clearance, are strongly associated with patient outcomes in both adult and pediatric critically ill patients [1,6,15,16]. In patients admitted to the pediatric cardiac intensive care unit (CICU), increased blood lactate level has been associated with significant morbidity and mortality [17,18]. However, both the production and the clearance of lactate can be impacted by several factors, which limit its interpretation. Lactate production can be increased by alternative mechanisms frequently encountered in intensive care, such as hyperglycemia or beta-receptor stimulation [19,20,21,22]. Both hyperglycemia and the adrenergic stimulation of beta-2 receptors will lead to excess pyruvate production at the mitochondrial level [20,23,24,25]. Part of this excess pyruvate will be diverted to lactate production. Reduced lactate clearance, as well as washout phenomena’ (temporary increase in serum lactate level once sufficient capillary blood flow has been reestablished), can impact serum lactate level even in the absence of tissue hypoxia.

Given the aforementioned limitations in the interpretations of conventional tissue oxygenation markers, supplementary tools to assess the hemodynamic status of critically ill patients are needed [26,27]. Carbon dioxide (CO_2_)-derived parameters may represent a potentially valuable addition to serum lactate level and ScVO_2_ when it comes to the evaluation of tissue perfusion and hypoxia, as underlined by the recent recommendation on hemodynamic monitoring adult experts when lack of data prevented pediatric experts from giving such recommendations [28,29]. The simplicity of CO_2_-derived parameters measurement at the bedside makes them an attractive tool to guide resuscitation in the clinical setting.

The purpose of this review is to describe currently available CO_2_-derived parameters and their physiological underpinnings, as well as their potential clinical applications in CICU.

## 2. CO_2_ Metabolism

CO_2_ is produced by cellular metabolism (VCO_2_) under both aerobic and anaerobic conditions. Under aerobic metabolism, CO_2_ is produced by the mitochondrial metabolism as a by-product of substrate oxidation. In the case of anaerobic metabolism, CO_2_ is also produced by bicarbonate buffering of H^+^ derived from lactic acid production and ATP hydrolysis. Carbon dioxide diffuses from the intracellular to the extracellular compartment. Once in the blood, CO_2_ transportation occurs in three forms: dissolved, as bicarbonate after the reaction of CO_2_ with H_2_O in red blood cells, and finally, as carbamino compounds within circulating proteins, particularly hemoglobin. The dissolved fraction of CO_2_ is in equilibrium with the partial pressure of CO_2_ (PCO_2_) according to Henry’s law of gas solubility, depending on the gas solubility, the atmospheric pressure, and P CO_2_ itself. The majority of CO_2_ is transported in the blood as bicarbonate. As CO_2_ diffuses nearly freely in red blood cells, it reacts with H_2_O to form carbonic acid (H_2_CO_3_). In turn, H_2_CO_3_ will dissociate to form both bicarbonate (HCO_3_^−^) and H^+^. Eventually, H^+^ will be buffered by hemoglobin, and HCO_3_^−^ exits the red blood cell. A small proportion of CO_2_ is transported as carbamino compounds that are linked to proteins, particularly to hemoglobin.

The proportion of diluted CO_2_, bicarbonate, and protein bounded CO_2_ varies between the arterial and venous compartments. In arterial blood, bicarbonate accounts for 90% of total CO_2_ content, while 5% is dissolved and 5% is bound to proteins. In venous blood, only 70% of the total CO_2_ content corresponds to bicarbonate, while 10% is dissolved, and a carbamino compound contributes 20%. The total content of CO_2_ (CCO_2_) in the blood under physiological conditions equals the dissolved CO_2_ + the bicarbonate (HCO_3_) + the CO_2_ linked to hemoglobin (R-NH_2_-CO_2_).

The content of CO_2_ (CCO_2_) is dependent on several variables and can be precisely calculated. According to the Douglas formula [30], the CCO_2_ could be determined using the content of CO_2_ in the plasma, the blood pH, and temperature:

Plasma CCO_2_ = 2.226 × S × plasma PCO_2_ × (1 + 10^pH-pK’^)

S = 0.0307 + [0.00057 × (37 − Temp)] + [0.00002 × (37 − Temp)^2^]

pK’ = 6.086 + [0.042 × (7.4 − pH)] + [(38 − Temp) × (0.00472 + (0.00139 × (7.4 − pH))]

For the final calculation, the hemoglobin level, the saturation in oxygenation, and the total amount of CO_2_ in the plasma are used:

CCO_2_ = Plasma CCO_2_ × [1 − [0.0289 × [Hb]/[[3.352 − 0.456 × SpO_2_] × [8.142 − pH]]]

The complete formula for the calculation of CCO_2_ gives the opportunity to appreciate the complexity of factors that are impacting the transport and content of CO_2_. However, despite its accuracy, this formula is extremely difficult to use at the bedside due to its computational complexity. Importantly, CO_2_ partial pressure (PCO_2_) is easier to obtain at the bedside, and a relationship exists between both CCO_2_ and PCO_2_ [31,32]. This relationship can be defined by the following equation PCO_2_ = *k* × CCO_2_, where *k* is a constant. This relationship follows a curvilinear curve with a near-linear relationship only in physiological PCO_2_ [31,32,33]. This nearly linear relation window has permitted the use of PCO_2_ in place of CCO_2_ in multiple clinical studies and expert opinion reports [26,28,34,35,36,37]. However, outside those ranges, it could be recommended to use CCO_2_ as the PCO_2_ will mostly divert significantly from the PCO_2_. The curvilinear relationship is directly impacted by the complex interaction of numerous variables such as the blood pH and temperature, the dissolved CO_2_, the CO_2_ bound to hemoglobin, and the CO_2_ as bicarbonate, as it is suggested by the multiple variables incorporated in the complete calculation of CCO_2_. The relationship between both CCO_2_ and pCO_2_ could be shifted by four factors: PO_2_, plasma pH and temperature, as well as hemoglobin levels. Those factors impact the *k* constant, with *k* increasing in case of a rise in PO_2_, acidosis, temperature, and hemoglobin level. Of special importance is the impact of oxygen on the content of CO_2_ through the Haldane effect. The Haldane effect describes the ability of hemoglobin to carry, at any PCO_2_, increased amounts of CO_2_ in the deoxygenated state compared to the oxygenated state [38]. Consequently, as blood enters the systemic microcirculation and releases O_2_, the CO_2_-carrying capacity increases so that the blood may remove the excess CO_2_. On the contrary, as blood enters the pulmonary circulation and binds O_2_, the CO_2_-carrying capacity decreases, thus facilitating CO_2_ removal from the lungs. In light of all the factors impacting the relationship between CCO_2_ and PCO_2_, we would argue for preferentially using CCO_2_ or, in case the PCO_2_ is used, it seems crucial that the clinicians understand the limitations of such approximation [38,39,40].

## 3. Relation between CO_2_ and Cardiac Output

### 3.1. Macrocirculation and Cardiac Output

Circulating CO_2_ is slightly higher in the venous compartment as a result of aerobic production of CO_2_ in tissues and alveolar elimination, thus creating a CO_2_ gradient between the venous and the arterial compartments. This difference will also be present under anaerobic circumstances due to the non-aerobic production of CO_2_. The increase in CO_2_ on the venous side will create an obligatory difference between the arterial and the venous blood, which could be estimated using the Fick equation.

In accordance with the Fick principle, the production of CO_2_ (VCO_2_) could be described as follows:

VCO_2_ = Cardiac Output (CO) × ΔCCO_2_

where ΔCCO_2_ is the difference in CCO_2_ between the venous and arterial compartments (CvCO_2_-CaCO_2_).

As mentioned above, PCO_2_ and CCO_2_ show a near-linear relationship at physiological ranges. Consequently, PCO_2_ values may be regarded as surrogate measures for CCO_2_ at the bedside.

A modified Fick equation can be obtained by substituting PCO_2_ with CCO_2_:

ΔPCO_2_ = (*k* × VCO_2_)/CO

**Figure 1:**
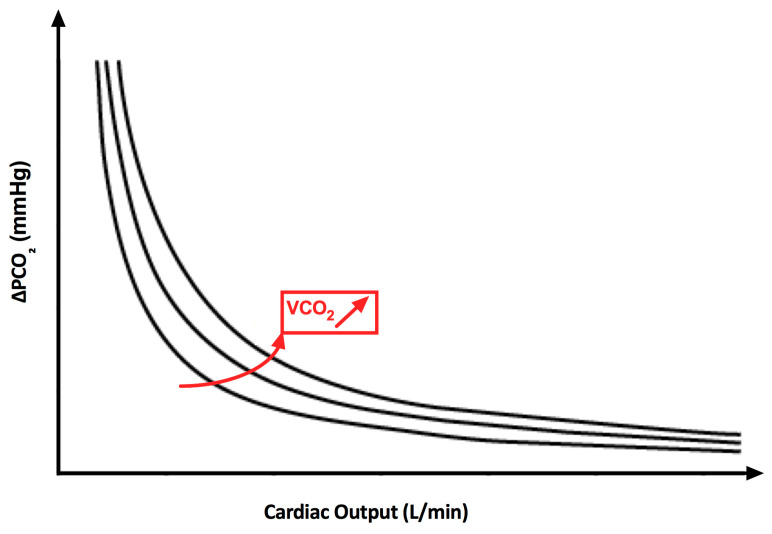
Relation between cardiac output and ΔPCO_2_. Inverse relation between cardiac output and ΔPCO_2_. Reduction of cardiac output is associated with an increase in ΔPCO_2_, initially slow, which become exponential at very low cardiac output. Modification of CO_2_ production (VCO_2_) shift the curves to the right and upward.

This equation shows the inversely proportional relationship between CO and ΔPCO_2_ (Figure 1).

The ability of ΔCCO_2_ and ΔPCO_2_ to monitor CO is unique and of primary importance. In shocked patients, the measurement of CO is a key element in the evaluation of the hemodynamic status of the patient as well as for the assessment of the appropriateness of the implementation of therapeutic measures. Although measuring CO in PICU patients is theoretically possible, the methods currently available are burdened by some limitations, which make their use in clinical practice sometimes difficult [41].

The gold standard of CO estimation is cardiac echocardiography through the measurement of the left ventricular outflow tract (LVOT) diameter and the aortic velocity time integral [42]. Nonetheless, the correct implementation of cardiac echography and its interpretation requires highly specialized training, and it may be difficult to perform in patients with congenital cardiac malformations. Other methods using Doppler technologies are available, such as Ultrasound Cardiac Output Monitor (USCOM™, Sydney, NSW, Australia) or transesophageal Doppler [43,44,45]. These devices have some drawbacks as they use pre-established LVOT diameters, and the comparison of CO values with thermodilution methods has yielded conflicting results [45]. Thermodilution methods use the change in temperature of the circulating blood after a cold saline bolus to estimate blood flow. Using either a pulmonary catheter or a femoral catheter, CO could be precisely monitored. Some catheters, such as the PICCO™ device, can continuously monitor CO. However, despite very promising data on the applicability and validity of the CO measure devices, the invasiveness and relative difficulties of use probably limit their use in the setting of clinical trials [46,47,48,49]. A more recent tool for CO measurement is represented by electrical bioimpedance. This technology is based on the detection of electrical resistance changes in electrical resistance at the level of the thorax skin caused by cardiac stroke volume. The reliability of this method is still under scrutiny [50]. Again, the presence of a congenital cardiac anomaly and intracardiac shunts further complicate the interpretation of CO measurements and represents an important limitation of the aforementioned techniques.

The fact that ΔPCO_2_ and ΔCCO_2_ only depend on CO under stable VO_2_ conditions makes its use in the ICU setting a reliable way to estimate the adequacy of CO with respect to tissue metabolic demand [51]. The relationship between ΔPCO_2_ and CO has been studied in previous reports. A progressive decrease in ΔPCO_2_ in parallel to an increase in CO in patients receiving escalating doses of dobutamine has been described [52,53]. Moreover, experimental models show that ΔPCO_2_ remains stable under hypoxic conditions, and low DO_2_ provides a normal and stable CO [54]. It is important to underline that, despite previous reports, ΔPCO_2_ does not change in the case of cellular hypoxia [55,56,57]. In fact, the stagnation of CO_2_ in tissues caused by the absence of sufficient blood flow but not hypoxia was responsible for the increase in ΔPCO_2_ in patients after cardiac arrest [58]. Clinicians might therefore disentangle low CO (low ScVO_2_ and elevated ΔPCO_2_) from cellular hypoxia in the presence of normal CO (low ScVO_2_ and normal ΔPCO_2_) [31,59]. Thus, unlike ScVO_2_, ΔPCO_2_ might play an important role as a further easy-to-use bedside tool to monitor CO regardless of the presence of hypoxemia.

### 3.2. Microcirculation and the Microhemodynamic

If ScVO_2_ can be helpful in assessing global hemodynamics with some caveats, it is not suitable for the evaluation of microcirculatory imbalances. Indeed, ScVO_2_ may exert normal values in case of microcirculation derangements because of a lower oxygen extraction [9,12,13]. In a state of shock, the increased heterogeneity of blood flow (that is, well-perfused vessels in close vicinity to non-perfused capillaries) along with a reduction in the functional capillary density may lead to an increase in ΔCCO_2_ regardless of CO [60].

Hypo-perfused areas will accumulate CO_2_ under anaerobic circumstances. The excess CO_2_ will then diffuse across well-perfused capillaries, and the ΔCCO_2_ will increase. The association between abnormal ΔPCO_2_ with altered microcirculatory blood flow has been demonstrated in septic shock patients [61,62,63,64]. ΔPCO_2_ should be regarded as a potential tool for the evaluation of microcirculatory perfusion abnormalities, even in the absence of low CO. Under such circumstances, the increase in CO might improve tissue perfusion [36,65,66,67].

### 3.3. Clinical Use of ΔCCO_2_ and ΔPCO_2_

In adult patients with septic shock, both ΔCCO_2_ and ΔPCO_2_ are associated with patient mortality [36,65,66,68]. The correlation between ΔCCO_2_ and ΔPCO_2_ with CO, along with the detection of microcirculation anomalies, could be the main reasons for such findings. Higher ΔPCO_2_ levels are also associated with both post-operative complications after cardiac surgery and mortality in general ICU patients [69,70,71,72]. Moreover, higher ΔPCO_2_ might indicate microcirculatory derangements and predict patient mortality in adult patients on ECMO [73]. A recent meta-analysis confirmed the association between higher ΔPCO_2_ and increased mortality in shocked ICU patients [74]. Importantly, ΔPCO_2_ plays no role as a marker of tissular hypoxia but rather as a marker of adequacy between CO_2_ production and CO. In fact, high-flow shocks should result in a decrease rather than an increase in ΔPCO_2_.

Several treatment algorithms based on the combined use of serum lactate, ScVO_2_, and ΔPCO_2_ as a marker of insufficient CO have been proposed [26,31,32]. Most of those algorithms suggest the use of ΔPCO_2_ with a cut-off value of 6 mmHg (0.8 kPa). Higher values will mostly indicate that CO is not able to meet global metabolic demands. In case of shock, a high ΔPCO_2_ could prompt clinicians to increase CO (fluid bolus and/or inotropes). In the absence of shock, a high ΔPCO_2_ might indicate a state of high oxygen demand and CO_2_ production. A normal ΔPCO_2_ might also indicate that CO is correct, while other determinants of oxygen delivery (SpO_2_, hemoglobin) should possibly be improved in the presence of anaerobic metabolism (increased lactate and/or VCO_2_/VO_2_ ratio). Many of the available algorithms propose the use of both ScVO_2_ and ΔPCO_2_ to help clinicians to identify anemia, hypoxemia, or low CO as possible causes of tissue hypoxia. To our knowledge, though, none of those algorithms have been clinically validated.

Studies in the pediatric population are scarce and showed promising but conflicting results. A recent pediatric study in children with septic shock failed to detect a clear association between higher ΔPCO_2_ and CO measures [75].

Most of the studies on ΔPCO_2_ have been conducted in the CICU setting. A study from Furqan et al. suggests a possible association between higher ΔPCO_2_ and low ScVO_2_ after pediatric cardiac surgeries [76]. In this study, normal ScVO_2_ was sometimes accompanied by increased ΔPCO_2_. Unfortunately, the association of normal ScVO_2_ and low ΔPCO_2_ with clinical outcomes was not explored.

We found three studies looking at the possible association between increased ΔPCO_2_ and unfavorable outcomes in the immediate post-operative period in children after cardiac surgery has been recently studied (Table 1) [35,77,78]. Two articles reported an association between higher ΔPCO_2_ and poor outcome, defined as a composite variable including death, cardiac arrest, ECMO requirement, unplanned surgical reinterventions, and elevated inotropic score [35,78]. On the contrary, Akamatsu et al. could not find such an association [77]. Differences in the study population, as well as the use of the PCO_2_ variable used in the analysis (continuous vs. dichotomous), might explain these conflicting results. Although encouraging, current literature on the relation between CO_2_-derived parameters and clinical outcomes in ICU is not yet conclusive. As ΔPCO_2_ is directly linked to the CO, a more immediate and precise outcome, such as the duration of inotrope/vasopressor support or the need for mechanical circulatory support, may be more suited.

### 3.4. Production of CO_2_ and O_2_ Consumption (VCO_2_/VO_2_ Ratio)

Oxygen consumption (VO_2_) and CO_2_ production (VCO_2_) are directly proportional to CO. Under aerobic steady-state conditions, the ratio between VO_2_ and VCO_2_ (VCO_2_/VO_2_ ratio) varies between 0.7 and 1, depending on the main metabolic substrate used for oxidative metabolism [79,80]. Under aerobic conditions, VCO_2_ should not exceed O_2_ availability; therefore, the ratio should not exceed one. However, under anaerobic conditions, during circulatory shock, both VO_2_ and VCO_2_ are globally reduced. However, due to buffering of cations (H^+^) by bicarbonate, a small production of CO_2_ remains [81]. This “anaerobic CO_2_ production” would result in a relative rise of VCO_2_ in comparison to VO_2_. As a result, under anaerobic metabolism and dysoxia, the VCO_2_/VO_2_ ratio will be higher than one [33].

The VCO_2_/VO_2_ ratio can be calculated at the bedside.

According to the Fick principle:

VO_2_ = CO × (CaO_2_ − CvO_2_)

and

VCO_2_ = CO × (CvCO_2_ − CaCO_2_)

The ratio is, therefore, equal to CvCO_2_ − CaCO_2_/CaO_2_ − CvO_2_. As mentioned above, at usual physiologic ranges, CvCO_2_ − CaCO_2_ can be replaced by ΔPCO_2_.

It gives the final equation:

VCO_2_/VO_2_ = ΔPCO_2_/CaO_2_ − CvO_2_

ΔPCO_2_, CaO_2_, and CvO_2_ are either directly accessible or easily calculated at bedside (CaO_2_ and CvO_2_ only need hemoglobin and oxygen saturation for their calculation).

The VCO_2_/VO_2_ ratio can identify anaerobic metabolism, and it has shown to be closely correlated with lactate levels, the usual dysoxia marker [33,39]. Moreover, the use of the VCO_2_/VO_2_ ratio might represent a possible alternative to lactate levels in specific situations that are frequently encountered in the ICU setting, such as hyperglycemia or catecholamine-induced hyperlactatemia.

### 3.5. Clinical Use of VCO_2_/VO_2_ Ratio

The VCO_2_/VO_2_ ratio has been less studied than ΔCO_2_. The princeps study by Mekontso-Dessap et al., carried out nearly 20 years ago, showed the role of the VCO_2_/VO_2_ ratio as a mortality marker in adult septic shock [66]. More recently, Ospina-Tascon also described the same relation, again in patients with septic shock [39]. Interestingly, even in patients with a normal lactate level, increased VCO_2_/VO_2_ ratio was strongly associated with a worse outcome. Other reports described the link between the VCO_2_/VO_2_ ratio and patients’ outcome both in septic shock and after cardiac surgery. Interestingly, both Ospina-Tascon et al. and He et al. showed an association between the VCO_2_/VO_2_ ratio and outcome in shocked patients with normal ScVO_2_ [39,82]. These findings underline the role of more advanced indices of tissue hypoxia when ScVO_2_ levels are within normal ranges, possibly signaling low O_2_ extraction and/or capillary perfusion disturbances [64,83]. This ratio might provide important prognostic information, especially in case of persistent high values and normalization of lactate levels after initial resuscitation.

The VCO_2_/VO_2_ ratio has also been described as a possible tool for the prediction of fluid responsiveness. In patients with a hypotensive episode, only those with an elevated VCO_2_/VO_2_ ratio showed a rapid increase in VO_2_ after the fluid challenge [65,84].

As with the ΔCO_2_, several algorithms propose the use VCO_2_/VO_2_ ratio in the initial patient assessment as a sign of tissue dysoxia. As for lactates, an elevated VCO_2_/VO_2_ ratio > one indicates anaerobic metabolism and should prompt a complete hemodynamic assessment of the patient and the possible implementation of rapid therapeutic measures to restore aerobic metabolism. On the other hand, a VCO_2_/VO_2_ ratio < one could also point to hyperglycemia or adrenergic stimulation as possible alternative causes of elevated lactate levels. Several cut-off values have been proposed to predict unfavorable outcomes, varying from 1.2 to 1.6 [31,37,85]. Unfortunately, as for the ΔPCO_2_, none of those algorithms has been validated in the clinical setting.

To the best of our knowledge, only one study looked at the VCO_2_/VO_2_ ratio in the pediatric population. Xu et al. studied the impact of elevated increased VCO_2_/VO_2_ ratio on acute kidney injury after pediatric cardiac surgery [86]. As blood is diverted from the kidneys in the case of shock, the authors suggest that an increased VCO_2_/VO_2_ ratio might represent a sign of anaerobic metabolism at the kidney level and indicate a possible kidney injury in CICU patients [87]. Despite the lack of clear evidence at the moment, these results suggest that VCO_2_/VO_2_ ratio may possibly help detect patients at risk of end-organ lesions in the context of anaerobic metabolism.

## 4. Implications for Research

Future studies are warranted to establish reference values of ΔPCO_2_ in CICU and PICU. Currently, reference values are derived from adult patients with septic shock. However, those values may differ significantly in children, especially in the cardiac population after a cardio-pulmonary bypass, where alterations of microcirculation may increase the ΔPCO_2_ value without macrohemodynamic disturbances. Our experience confirms current pediatric literature describing large ΔPCO_2_ regardless of clinical outcomes. Multicenter prospective cohort studies are also needed to better define the relationship between ΔPCO_2_.and cardiac output in the case of LCOS. The description and validation of the impact of CO_2_-derived parameters in patient management, both adult and pediatric patients, are highly needed. Whether the introduction of CO_2_-derived parameters into clinical algorithms may improve patients’ outcomes is unknown.

## 5. Conclusions

Despite the many limitations and the lack of robust data, CO_2_-derived parameters such as ΔPCO_2_ and VCO_2_/VO_2_ ratio represent valuable markers of hemodynamic derangements from macrocirculation to microcirculation. Moreover, unlike traditional markers of cardiac output, they seem to be reliable in specific situations commonly encountered in the post-cardiac surgery setting, such as hyperglycemia and catecholamine use. Their integration with classical markers of hypoperfusion into treatment algorithms holds the promise of adding substantial information that might help refine the management of patients suffering from shock in the adult and in the pediatric population alike. Further studies are needed to clearly define the role of those attractive tools in guiding resuscitation in the clinical setting.

## Figures and Tables

**Table 1:** Pediatric study looking ΔCO_2_ as a tool in resuscitation.

	Insom et al. [78] Cardiol Young 2021	Rhodes et al. [35] PCCM 2017	Akamatsu et al. [77] PCCM 2017
N patients	40 patients	139 patients	114 patients
Age (days/months)	Median 215 days (range 3–5600)	Median 12 days (IQR 6–38)	Median 15.5 months (IQR 7–34)
Population	CICU	CICU	CICU
Outcome measured	Composite outcome − VIS > 15 − CICU LOS > 5 days	Composite outcome: − IS > 15 − Mortality − Cardiac arrest − ECMO within 48 h − Reintervention	− Mecanical ventilation duration − Mortality
∆PCO_2_ measured (mmHg)	Median 9 mmHg (range 1–25)	Median 5.9 mmHg (IQR 3.8–9.2)	Not reported ∆PCO_2_ analyzed as a dichotomous variable >6 mmHg or <6 mmHg
Association ∆PCO_2_-outcome	Significant association OR 1.13 (95% CI 1.01–1.35)	Significant association − composite outcome OR 1.3 (95% CI 1.1–1.45) − Mortality OR 1.2 (95% CI 1.07–1.31)	No significant association
Commentary	Higher values of ∆PCO_2_ are associated with more complex clinical course.	Underline role for ∆PCO_2_ monitoring in CICU Suggest an association between ∆PCO_2_ and outcome.	Population separated into 2 groups: ∆PCO_2_ > 6 mmHg or <6 mmHg and no difference between both groups.

CICU, cardiac intensive care unit.

## Data Availability

Not applicable.
